# Dysphagia for medication in Parkinson’s disease

**DOI:** 10.1038/s41531-022-00421-9

**Published:** 2022-11-12

**Authors:** Bendix Labeit, Elijahu Berkovich, Inga Claus, Malte Roderigo, Anna-Lena Schwake, Dvora Izgelov, Dorit Mimrod, Sigrid Ahring, Stephan Oelenberg, Paul Muhle, Verena Zentsch, Fiona Wenninger, Sonja Suntrup-Krueger, Rainer Dziewas, Tobias Warnecke

**Affiliations:** 1 Department of Neurology with Institute of Translational Neurology, University Hospital Muenster, Muenster, Germany; 2 Institute for Biomagnetism and Biosignal Analysis, University Hospital Muenster, Muenster, Germany; 3 Clexio Biosciences LTD, Jerusalem, Israel; 4 Department of Neurology and Neurorehabilitation, Klinikum Osnabrueck – Academic teaching hospital of the WWU Muenster, Osnabrueck, Germany

**Keywords:** Parkinson's disease, Neurodegenerative diseases

## Abstract

Dysphagia is common in Parkinson’s disease (PD) and is assumed to complicate medication intake. This study comprehensively investigates dysphagia for medication and its association with motor complications in PD. Based on a retrospective analysis, a two-dimensional and graduated classification of dysphagia for medication was introduced differentiating swallowing efficiency and swallowing safety. In a subsequent prospective study, sixty-six PD patients underwent flexible endoscopic evaluation of swallowing, which included the swallowing of 2 tablets and capsules of different sizes. Dysphagia for medication was present in nearly 70% of PD patients and predicted motor complications according to the MDS-UPDRS-part-IV in a linear regression model. Capsules tended to be swallowed more efficiently compared to tablets, irrespective of size. A score of ≥1 on the swallow-related-MDS-UPDRS-items can be considered an optimal cut-off to predict dysphagia for medication. Swallowing impairment for oral medication may predispose to motor complications.

## Introduction

Oropharyngeal dysphagia (OD) is common in Parkinson’s disease (PD) and not only reduces patients’ quality of life, but leads to serious complications such as malnutrition, and aspiration pneumonia, thereby increasing mortality^1^. Since swallowing cannot be reliably assessed by clinical examination alone and PD patients may not notice symptoms of dysphagia, instrumental diagnostics are essential to reliably detect OD^2^. Both, Flexible Endoscopic Evaluation of Swallowing (FEES) and Videofluoroscopic Swallow Study (VFSS) are considered the diagnostic gold standards^3,4^. However, in PD patients with fluctuating symptoms, FEES has distinct advantages, as long examination protocols and repeated examinations are easily possible since there is no radiation exposure involved.

In addition to OD during food intake, studies relying on self-reporting suggest that PD patients often experience difficulty swallowing their medications^5–7^. This frequently leads to patients or caregivers modifying the oral dosage form, e.g., dividing, dissolving, or crushing tablets and opening capsules, or even omitting the medication altogether^8–13^. Dosage form modifications can adversely affect both pharmacodynamics and -kinetics and result in inadequate efficacy, adverse events or even death^11,13–16^. Therefore, it is not surprising that individual case reports and case series link dysphagia during medication swallowing in PD to lack of medication efficacy or to motor fluctuations such as delayed on-phenomena^17–19^. However, there have been few prospective studies systematically investigating dysphagia for medication in PD using instrumental diagnostics^19,20^. Moreover, in these previous studies, dysphagia for medication was classified in a binary manner (e.g., present vs. absent), thus lacking a differentiated analysis with associations to motor complications and to dysphagia for food and liquid.

Therefore, the aims of this study were (1) to investigate the relationship between dysphagia for medication and motor complications, (2) to examine the prevalence and severity of dysphagia for medication depending on the type of tablet or capsule and in relation to OD for food and liquid, and (3) to investigate the predictive value of questions relevant to oropharyngeal dysfunction in the Movement-Disorder-Society-Unified-PD-Rating-Scale (MDS-UPDRS) in relation to dysphagia for medication. First, a two-dimensional and ordinal classification of dysphagia for medication was established based on a retrospective analysis. This classification was then used to investigate the swallowing of tablets and capsules of different sizes and shapes in 66 PD patients in a prospective study using FEES.

## Results

### Classification of dysphagia for medication

**Fig. 1:**
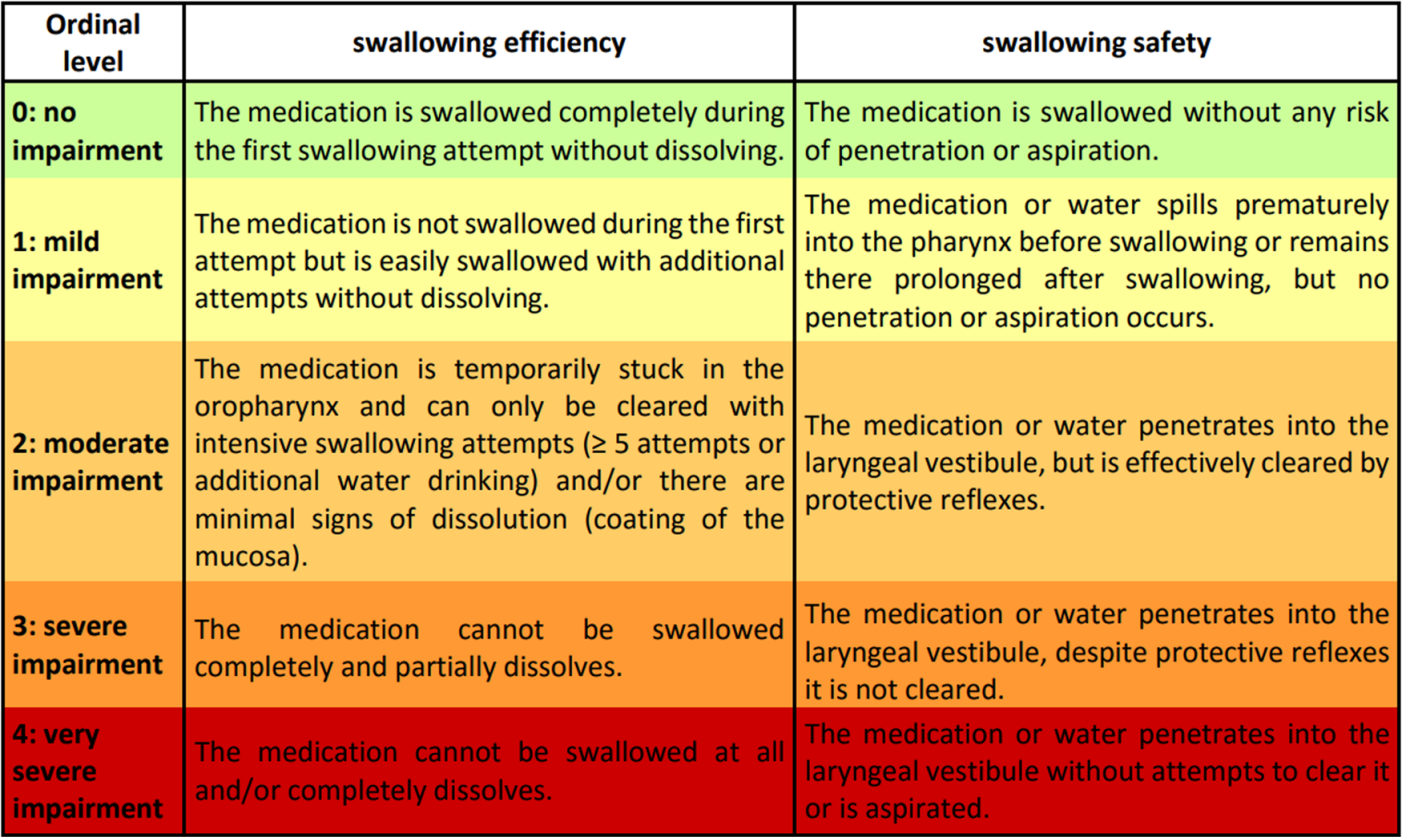
Classification of Dysphagia for Medication. Illustration of the 5-point ordinal scales of impairment in swallowing efficiency and swallowing safety.

Two different dimensions of swallowing impairment in medication swallowing were identified: (1) Swallowing efficiency: The ability to transport the tablet or capsule completely (without any dissolution) from the oral cavity into the esophagus with a single swallow. (2) Swallowing safety: The ability to fully protect the airway during swallowing without risk of aspiration or penetration of medication or water. The impairment severity levels which were identified for these two impairment dimensions are illustrated in Fig. 1. Interrater reliability was *κ* = 0.89 (*p* < 0.001) for swallowing efficiency and *κ* = 0.86 (*p* < 0.001) for swallowing safety, suggesting high reliability for both impairment dimensions.

### Clinical characteristics and prevalence of dysphagia

66 subjects were enrolled in the study. All patients fully completed the FEES protocol. Table 1 illustrates demographic and clinical characteristics of the cohort as well as prevalence and severity of OD for food and liquid and overall dysphagia for medication (Methods-section “Swallowing-assessment”) across all tablets and capsules. Both forms of dysphagia were common in PD patients and occurred with at least mild impairment in ~70% of patients.

**Table 1:** Demographics and prevalence of oropharyngeal dysphagia for food and liquid and dysphagia for medication: *SD* standard deviation, *OD* oropharyngeal dysphagia, *PAS* Penetration Aspiration Scale.

Parameter	Value
Mean age ± SD in years	68.4 ± 8.8
Sex m/f	44/22
Hoehn & Yahr, *n* (%)	
2	29 (43.9%)
2,5	10 (15.2%)
3	17 (25.8%)
4	9 (13.6%)
5	1 (1.5%)
OD for food and liquid, *n* (%)	
No sign	20 (30.3%)
Mild	38 (57.6%)
Moderate	5 (7.6%)
Severe	3 (4.5%)
PAS 1	58 (87.9%)
PAS 2	2 (3.0%)
PAS 3	4 (6.1%)
PAS 6	1 (1.5%)
PAS 7	1 (1.5%)
Overall dysphagia for medication, *n* (%)	
No signs	22 (33.3%)
Mild	20 (30.3%)
Moderate	15 (22.7%)
Severe	3 (4.5%)
Very severe	6 (9.1%)

### Dysphagia for medication and motor complications

A significant linear regression equation was found (F[2,59] = 4.3, *p* = 0.017) to predict motor complications according to the summarized score of the MDS-UPDRS-part-IV with age and the dysphagia for medication score (Methods-section “Swallowing-assessment”) as independent variables. *R*-square was 0.128, indicating that ~13% of the variance was explained by the model. Dysphagia for medication significantly predicted motor complications (beta-coefficient: 0.5; *p* = 0.006) whereas age was not a predicting variable (beta-coefficient: −0.1; *p* = 0.332). Because of the violation of the homoscedasticity of the residuals and the normal distribution of the residuals, additional wild bootstrapping with 2000 iterations was performed in which dysphagia for medication also predicted motor complications (beta-coefficient: 0.5; *p* = 0.003).

### Dysphagia for medication depending on the type of tablet or capsule

Table 2 illustrates the frequency of impaired swallowing efficiency and swallowing safety as well as the mean values of subjective difficulty and anxiety during swallowing depending on the type of tablet or capsule. The Friedman’s-test revealed a difference in swallowing efficiency [*χ*^2^(3) = 14.6, *p* = 0.002], subjective difficulty [*χ*^2^(3) = 26.5, *p* < 0.001] and subjective anxiety [*χ*^2^(3) = 19.7, *p* < 0.001] depending on the type of tablet or capsule. However, the Friedman’s-test for swallowing safety [*χ*^2^(3) = 1.6, *p* = 0.653] did not suggest differences between the type of tablets or capsules. The post-hoc analysis of pairwise comparisons for swallowing difficulty further revealed more severe difficulty with the large tablet vs. the small capsule and vs. the small tablet (Table 2). The post-hoc analyses of pairwise comparisons for swallowing efficiency as well as subjective anxiety did not reveal significant results. No statistical difference was detected in swallowing efficiency, safety, subjective difficulty or subjective anxiety per the large capsule vs. any other dosage form. However, the raw data in detail suggest a tendency for higher swallowing efficiency of capsules compared with tablets, and greater anxiety when swallowing larger capsules and tablets compared with small products.

**Table 2:** Number and percentage of cases with impaired swallowing efficiency, swallowing safety, mean subjective difficulty, mean subjective anxiety as well as *p* value of the Friedman’s-test depending on the oral dosage form and the Bonferroni adjusted *p* values in the post hoc analysis comparing the different oral dosage forms.

Swallowing parameter	Large capsule	Small capsule	Large tablet	Small tablet	*p* value
Swallow efficiency, *n* (%)					0.002*
No signs	56 (84.8%)	58 (87.9%)	46 (69.7%)	48 (72.7%)	
Mild	4 (6.1%)	4 (6.1%)	11 (16.7%)	10 (15.2%)	
Moderate	6 (9.1%)	4 (6.1%)	5 (7.6%)	4 (6.1%)	
Severe	0 (0%)	0 (0%)	2 (3.0%)	1 (1.5%)	
Very severe	0 (0%)	0 (0%)	2 (3.0%)	3 (4.6%)	
Swallow safety, *n* (%)					0.653
No signs	48 (72.7%)	49 (74.2%)	44 (66.7%)	46 (69.7%)	
Mild	15 (22.7%)	13 (19.7%)	17 (25.8%)	15 (22.7%)	
Moderate	3 (4.5%)	3 (4.5%)	3 (4.5%)	4 (6.1%)	
Severe	0 (0%)	1 (1.5%)	1 (1.5%)	0 (0%)	
Very severe	0 (0%)	0 (0%)	1 (1.5%)	1 (1.5%)	
Subjective difficulty, mean ± SD	2.1 ± 2.5	1.3 ± 2.0	2.7 ± 2.7	1.7 ± 2.2	0.001*
Subjective anxiety, mean ± SD	1.0 ± 2.2	0.2 ± 0.8	0.4 ± 1.2	0.2 ± 0.7	0.001*
Oral dosage form comparison (post hoc; *p* value)	Swallow efficiency	Swallow difficulty	Swallow anxiety	swallow safety	
Large capsule vs. small capsule	>0.999	0.157	0.412	n.a.	
Large capsule vs. large tablet	0.726	0.591	>0.999	n.a.	
Large capsule vs. small tablet	>0.999	>0.999	0.591	n.a.	
Small capsule vs. large tablet	0.328	*0.001	>0.999	n.a.	
Small capsule vs. small tablet	0.634	>0.999	>0.999	n.a.	
Large tablet vs. small tablet	>0.999	*0.028	>0.999	n.a.	

### Dysphagia for medication depending on OD for food and liquid

The cross-tabulation of dysphagia for medication with OD for food and liquid and the PAS is presented in Table 3. There was a moderate correlation between the severity of OD for food and liquid and dysphagia for medication (Spearman’s rho correlation coefficient: 0.39; *p* = 0.001) and a weak correlation between the PAS and dysphagia for medication (Spearman’s rho correlation coefficient: 0.28; *p* = 0.023). However, 6 out of 9 subjects with severe or very severe dysphagia for medication showed only mild or no OD for food and liquid without penetration or aspiration.

**Table 3:** Cross-tabulation of the severity of oropharyngeal dysphagia for food and liquid and overall dysphagia for medication: *OD* oropharyngeal dysphagia, *PAS* Penetration Aspiration Scale.

		Overall dysphagia for medication					
	Severity, *n*	None	Mild	Moderate	Severe	Very severe	Total, *n*
OD for food and liquid	None	10	8	1	0	1	20
	Mild	12	9	12	1	4	38
	Moderate	0	2	2	1	0	5
	Severe	0	1	0	1	1	3
	PAS 1	22	17	13	1	5	58
	PAS 2	0	1	0	1	0	2
	PAS 3	0	1	2	0	1	4
	PAS 6	0	0	0	1	0	1
	PAS 7	0	1	0	0	0	1
	total, n	22	20	15	3	6	66

### Swallowing-MDS-UPDRS-items as predictor of dysphagia for medication

**Fig. 2:**
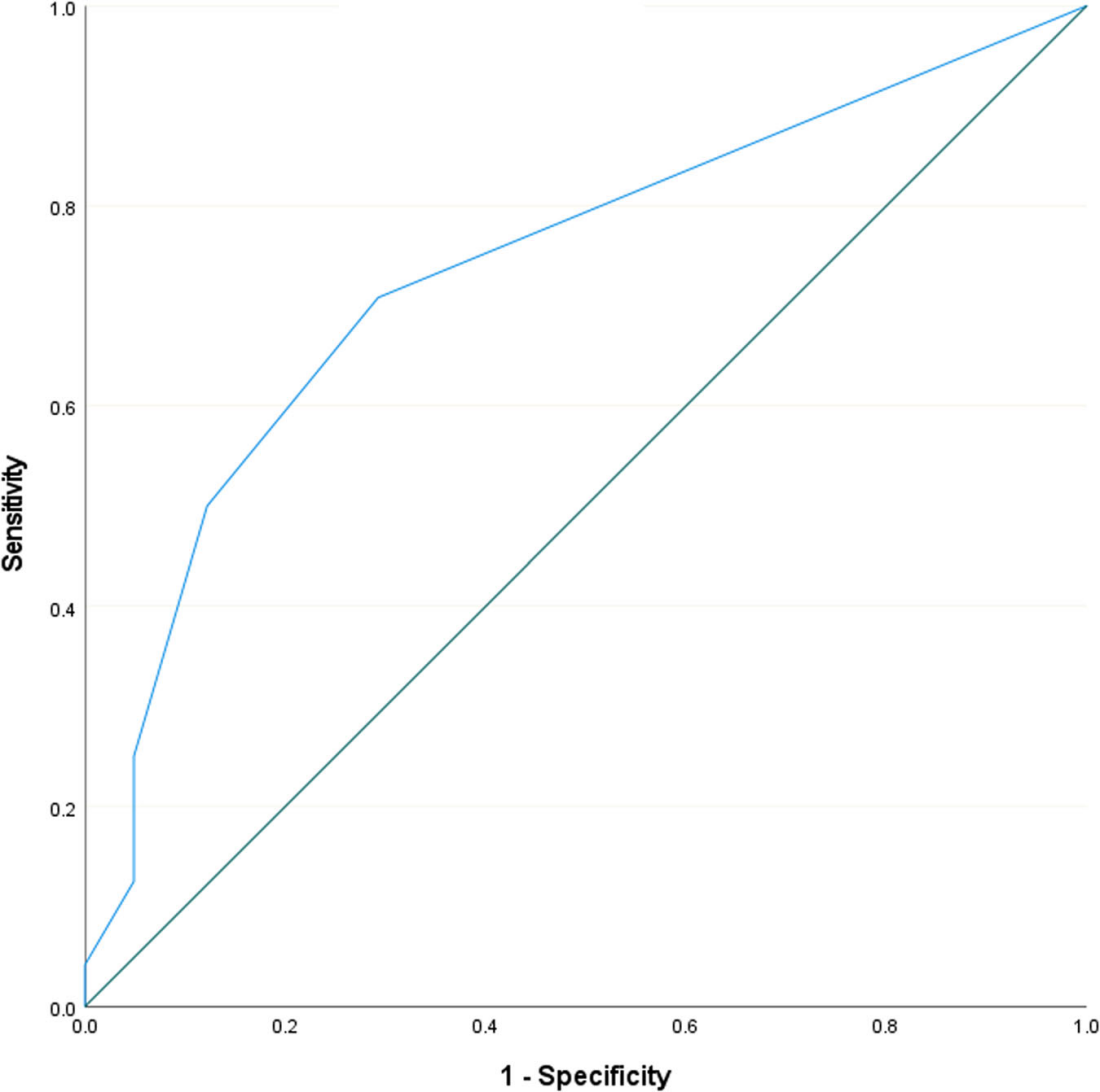
Predicting Dysphagia for Medication. Receiver operating characteristic (ROC) curve with the sum score of the 2 swallowing-related Unified Parkinson’s Disease Rating Scale items predicting at least moderate overall dysphagia for medication.

To predict at least moderate overall dysphagia for medication with the sum-score of the 2 swallowing-related MDS-UPDRS-items, a ROC-Analysis was performed. The ROC-analysis revealed a significant area under the curve (*p* = 0.001, AUC: 0.74) and the Youden-Index indicated that a score of ≥1 can be considered as optimal cut-off-value to predict moderate overall dysphagia for medication (sensitivity: 70.8%, specificity: 70.7%, positive predictive value: 58.6%, negative predictive value: 80.6%). Figure 2 illustrates the ROC-curve.

## Discussion

According to our study, two different dimensions, swallowing efficiency and swallowing safety, should be assessed when evaluating dysphagia for medication. Swallowing efficiency refers to the ability to transport the medication completely from the oral cavity into the esophagus. The proposed classification considers both the premature dissolution of the medication in the oropharynx and the number of swallows required for transport. Swallowing safety evaluates whether the airway is adequately protected during medication swallowing. This study implemented an ordinal classification and thus differs from the other 2 studies that investigated dysphagia for medication in PD using instrumental diagnostics, in which patients were binary divided into dysphagic and nondysphagic^19,20^. Fukae et al. reported medication residue after swallowing using FEES in 6 out of 9 PD patients with delayed-on phenomenon vs. no medication residue in 9 PD patients without delayed-on phenomenon^19^. Buhmann et al. studied the swallowing of 3 tablets with different sizes and shapes and 1 capsule in 118 PD patients using FEES. They reported that 85 subjects had no or only minor swallowing impairment. Minor impairment was defined as a transient medication residue in the oropharynx, which had to be noticed by the patient and cleared spontaneously or by additional water swallowing. Moderate or severe deficits were detected in 33 patients. Cases were rated as moderate when medication residue was not noticed by patients or could only be cleared insufficiently. Severe dysphagia was assigned if direct or indirect signs of aspiration were observed or if the medication could only be taken crushed or with puree^20^. Thus, aspects of swallowing efficiency such as medication residue or insufficient clearing and aspects of swallowing safety such as aspiration signs were also taken into account in these previous studies. However, swallowing efficiency and swallowing safety were not subdivided into separate dimensions but formed part of the binary categorization. Aspects of tablet dissolution, on the other hand, were not considered. In further studies on medication swallowing in different cohorts, VFSS was used to characterize dysphagia^21–26^. The major advantage of this method is that the esophageal phase can be assessed. Data from these studies, mainly from the 1980s suggest that a prolonged transit time and premature dissolution of the medication in the esophagus may occur^21–23,25^. Disadvantages of VFSS imaging are that only a limited number of swallows can be examined due to radiation exposure and that pharyngeal structures such as the coating of the mucosa with the dissolving medication can only be examined to a limited extent.

With regard to prevalence, our data show that dysphagia for medication is very common in PD at approx. 70% and has a comparable prevalence to OD for food and liquid. These prevalence data are higher than the previously reported studies by Buhmann et al with 28%^20^ and Fukae et al with 33%^19^. However, in Buhmann et al. only significant impairment was designated as abnormal. In Fukae et al. only medication residue and not impaired swallowing safety was analyzed.

With regard to motor symptoms, our data show a significant, albeit weak, association between dysphagia for medication and motor complications. The causes of motor complications in PD are not yet understood in detail and are probably heterogeneous^27^. Possibly, in addition to gastric motility disorders, dysphagia for medication may also be involved in reduced or fluctuating absorption of dopaminergic medication in the small intestine. Matching this, Fukae et al. demonstrated that tablet residue in FEES were associated with the delayed-on phenomenon in PD patients^19^. Further, case reports suggest an association of dysphagia for medication with motor fluctuations^17,18^. In addition, switching levodopa from oral to dispersible form has been shown to improve morning akinesia, delayed-on, and wearing-off phenomena in PD patients with swallowing impairment^6^. In contrast, Buhmann et al. did not find an association between dysphagia for medication and dopaminergic response, which was scored binary based on patient history^20^.

Impairment of swallowing efficiency significantly differed depending on the type of tablet or capsule whereas no relevant differences were observed for impairment of swallowing safety. Although post-hoc comparisons of swallowing efficiency did not further characterize the dependence on the type of tablet or capsule, the raw data suggest that capsules tend to be swallowed more efficiently than tablets. These results are in line with the findings of Buhmann et al. using FEES in PD. The authors reported a significant difference between the capsule, which was easiest to swallow, and the oval tablet, which was most difficult to swallow^20^. One possible reason for this finding could be that the capsule surface causes less static friction than the tablet surface. It also seems plausible that unlike tablets, capsules do not dissolve as quickly, but are more stable and remain intact longer. In this way, at least in milder cases of dysphagia, there is no premature dissolution of the medication. The size of the product, on the other hand, does not seem to have a relevant influence on the objective findings in instrumental diagnostics. In particular, the large capsule investigated in our study did not result in more severe impairments in objective swallowing diagnostics. A possible explanation in terms of swallowing efficiency could be that the relative surface area compared to weight is smaller for large products, resulting in lower static friction. Larger and heavier products might also trigger increased or more sufficient swallowing. In terms of swallowing safety, a larger product could increase sensory perception, thus enhancing sensorimotor feedback as a compensatory mechanism. This is supported by previous clinical studies that have shown that intact pharyngeal sensation is important for a physiological swallowing process^28,29^. The findings on medication size contrast with a Food and Drug Administration (FDA) guidance document that considers the size of tablets or capsules to be a possible risk factor for dysphagia for medication and therefore recommends the production of small products. At the same time, however, reference is made to the lack of data on objectively assessed oropharyngeal medication swallowing^30^.

When considering the subjective perception, the large tablet was perceived as more difficult to swallow than the small capsule and small tablet, while no significant difference in swallowing difficulty was detected for the large capsule vs. the other dosage forms or sizes. In addition, there tended to be a greater anxiety of swallowing larger products, although the post-hoc comparison did not reveal any significant differences between the individual dosage forms. In line with this, further studies show that patients in general subjectively prefer smaller preparations^31–33^. In contrast, another study suggests that both too small and too large preparations may be perceived as problematic^34^. A possible reason why size may be a decisive parameter for the subjective swallowing experience could again be the stronger sensory perception, which may be perceived as unpleasant.

The examination of the relationship between OD for liquid and food an dysphagia for medication revealed a moderate correlation. Thus, patients with OD for food and liquid are also at increased risk for dysphagia for medication. However, six out of 9 patients with at least severe dysphagia for medication had mild or no OD for food and liquid and showed no penetration and aspiration. Thus, it should not be assumed across the board that patients with only mild OD for food and liquid are also capable of swallowing medications. These results are consistent with those of Buhmann et al. The authors reported that half of the subjects with dysphagia for medication also had dysphagia for liquids, whereas the other half did not^20^. Therefore, we argue that it is useful to evaluate OD for food and liquid and dysphagia for medication separately. Then, based on the patient’s individual findings, an appropriate medication strategy can be found and, if necessary and possible, a switch to oral dispensable, liquid, subcutaneous, or continuous intestinal substitutes can be recommended.

Our data show that the swallowing-related items of the MDS-UPDRS can be used with moderate sensitivity and specificity to predict dysphagia for medication. Therefore, as soon as there is evidence of impairment in items 2.3 or 2.4, it may be appropriate to initiate a more detailed investigation of dysphagia for medication. Here, our results differ from those of Buhmann et al. where poor diagnostic indicators were obtained by questionnaire screening^20^.

In conclusion, dysphagia for medication is common in PD and can affect both swallowing efficiency, i.e., the ability to swallow medications completely, and swallowing safety, i.e., the ability to protect the airway. This study indicates that dysphagia for medication is involved in the development of motor complications. Both, large and small capsules tended to be swallowed more efficiently than tablets, whereas the safety of swallowing did not depend on the type of tablet or capsule. Dysphagia for medication should be evaluated independently of normal bolus OD, as the two moderately correlate, but may differ substantially in individual cases. If there is evidence of impaired swallowing or food intake in the MDS-UPDRS items 2.3 and 2.4, further diagnostic workup is advised, as this is a predictor of dysphagia for medication with moderate sensitivity and specificity.

There are several limitations that must be considered: The classifications used are based on expert consensus and have not yet been empirically validated. In particular, the weighting of the individual severity levels of dysphagia for medication and their variability within a subject have not yet been investigated, so this classification must be regarded as provisional and will require further validation and, if necessary, modification in the future. There were no events of complete bolus aspiration in our study. This must be taken into account with regard to the statements on swallowing safety, especially with regard to the analysis on the different tablets or capsules. It is possible that complete aspiration of large product might be more dangerous compared to small products, however, this is not reflected in our data due of the rarity of such events. In addition, the texture of the medication surface was not analyzed and there was no control group of healthy subjects.

## Methods

### Development of the classification of dysphagia for medication in PD

The classification was developed by an interdisciplinary team of neurologists (*n* = 2) and speech-language therapists (*n* = 2). Thirty selected FEES videos of PD patients with swallowing of medication, previously recorded for diagnostic reasons, were analyzed. In the first step, characteristic findings of impairment were collected that deviated from physiological medication swallowing. The latter was defined as complete swallowing of the medication during the first swallowing attempt without risk of aspiration. Subsequently, the described findings were examined with regard to different dimensions of impairment (that were assumed to occur independently of each other). Next, different levels of impairment severity were identified for each impairment dimension previously defined. In a final step, it was checked whether all levels of impairment could be clearly distinguished from each other. If this was not the case, the classification level was specified and more clearly demarcated from the other levels. In case of disagreement within the group, the discussion continued, and more video examples were viewed until a mutual consensus was reached.

### Patient cohort

Patients were prospectively enrolled between 13th April and 31st August 2021 at the University Hospital of Muenster. All patients were diagnosed with idiopathic PD according to the British Parkinson’s Society Brain Bank criteria, respective the revised MDS criteria^35,36^. Furthermore, as an inclusion criterion, a Hoehn and Yahr disease stage of ≥2 had to be present. Patients were excluded if they required a gastric feeding tube due to OD, if other conditions associated with OD were present, if oropharyngeal surgery had been performed within the past year, or if they received deep brain stimulation or continuous pump therapy. All patients who fulfilled the inclusion and none of the exclusion criteria were asked to participate in the study. None of the initially enrolled patients dropped out of the analysis. The study design was approved by the local ethics committee (Ethik-Kommission der Ärztekammer Westfalen-Lippe und der Westfälischen Wilhelms-Universität Münster). All participants gave written informed consent prior to study participation.

### Study design

**Fig. 3:**
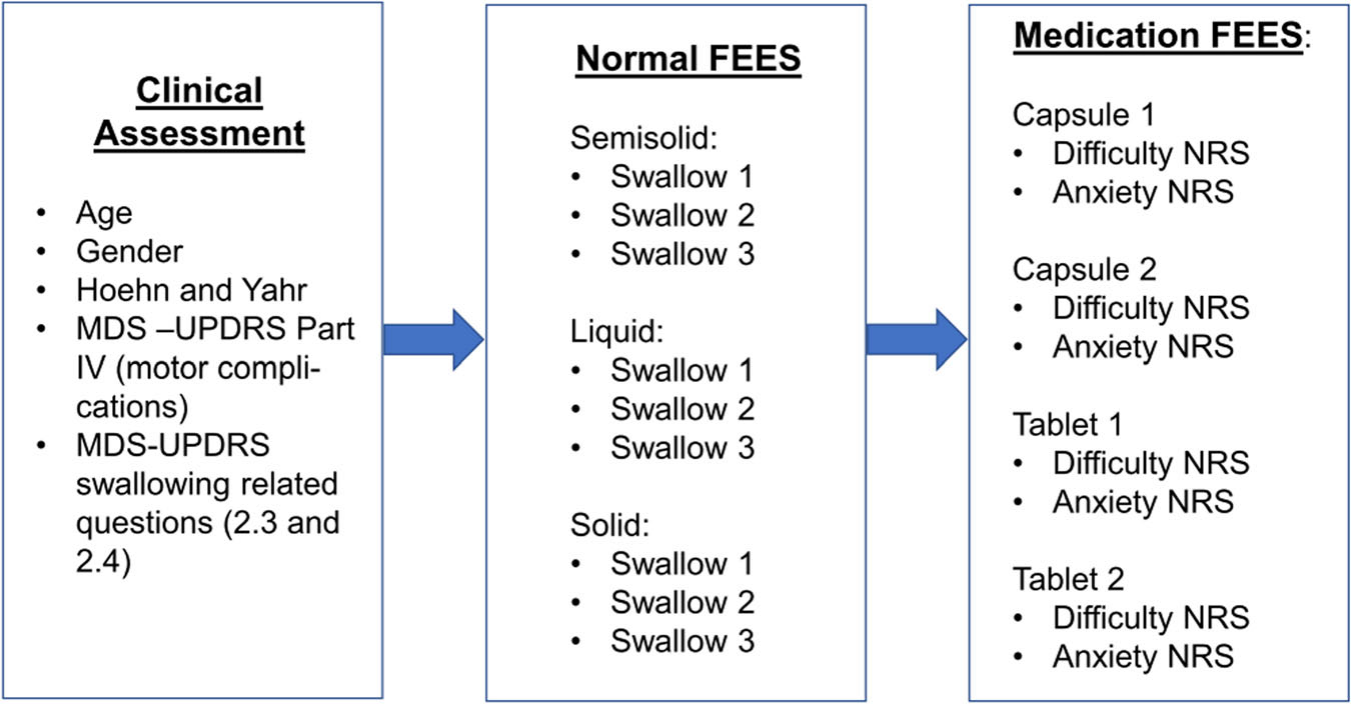
Illustration of the study design. FEES Flexible Endoscopic Evaluation of Swallowing, MDS-UPDRS Movement Disorder Society-sponsored revision of the Unified Parkinson’s Disease Rating Scale, NRS Numeric Rating Scale.

Patients were examined during the clinical “on-state”. First, demographic and clinical assessments including age, sex, Hoehn and Yahr stage, the MDS-UPDRS-Part-IV on motor complications and the swallowing related question items of the MDS-UPDRS-Part-II (2.3 on problems swallowing pills or eating meals and 2.4 on troubles handling food) were performed. Then, subjects underwent FEES, which assessed both swallowing of food and liquids and swallowing of placebo medication. Food and liquid swallowing consisted of three different food consistencies in the following order: 8 ml of green jelly (semi-solid), 5 ml blue-dyed liquid, and white bread (solid) with a size of approximately 3 × 3 × 0.5 cm. Each consistency was tested in 3 swallowing trials. Medication swallowing consisted of a total of 4 swallowing trials, starting with 2 placebo gelatin capsules in random order (large capsule: length: 29 ± 1 mm, width: 9.6 mm, weight: 1883mg ± 1%, manufacturer: Lonza; small capsule: length: 19.4 mm, width: 6.8 mm, weight: 300 mg, manufacturer: custom made, pharmacy University Hospital Muenster) and followed by 2 placebo tablets in random order (round and small tablet: diameter 10 mm, manufacturer: Winthrop Arzneimittel GmbH - PZN: 04997450; large and oval tablet: length 17 mm, width 8.2 mm, manufacturer: Fagron GmbH - PZN: 00921088). The placebo medication was handed along with a glass of water. After each medication swallowing trial, subjects were asked to rate subjective swallowing difficulty and subjective anxiety during swallowing on a Numeric Rating Scale (NRS) from 0 to 10. Figure 3 illustrates the study procedure.

### Swallowing assessment

#### OD for food and liquid

Swallowing impairment for food and liquid was classified according to the following ordinal scale as previously published^37–39^:

- No signs of dysphagia.
- Mild dysphagia: premature bolus spillage at least into the piriform sinus or/and pharyngeal residue > coating in at least 2 out of 3 swallowing trials of at least 1 consistency, but no signs of penetration or aspiration.
- Moderate dysphagia: penetration or/and aspiration in at least 1 swallowing trial of 1 consistency.
- Severe dysphagia: penetration or/and aspiration in at least 1 swallowing trial of more than 1 consistency.

Furthermore, Rosenbek’s Penetration Aspiration Scale (PAS)^40^ was determined, with the highest score reported for each patient during food and liquid swallowing.

#### Dysphagia for medication

Swallowing impairment for medication was rated according to the newly developed Classification of Dysphagia for Medication (Results-section “Classification of Dysphagia for Medication”). To determine interrater reliability, the classification was independently scored by a second rater in 15 random participants. Overall, the severity of dysphagia for medication was ordinally scaled based on the ratings of the Classification of Dysphagia for Medication (Results-section “Classification of Dysphagia for Medication”) as follows:

- no signs of dysphagia for medication.
- mild: signs of mild impairment of swallowing safety or/and swallowing efficiency in at least 1 of the tested medication trials.
- moderate: signs of moderate impairment of swallowing safety or/and swallowing efficiency in at least 1 of the tested medication trials.
- severe: signs of severe impairment of swallowing safety or/and swallowing efficiency in at least 1 of the tested medication trials.
- very severe: signs of very severe impairment of swallowing safety or/and swallowing efficiency in at least 1 of the tested medication trials.

In addition, an overall dysphagia for medication score was calculated for each patient. For this purpose, the numerical rating for the two dimensions of swallowing safety (0–4) and swallowing efficiency (0–4) (Results-section “Classification of Dysphagia for Medication”) were summarized for each of the 4 swallowing trials, resulting in a total score ranging from 0 to 32.

### Statistical analysis

#### Interrater reliability of the classification of dysphagia for medication

The interrater reliability of both sub-dimensions (swallowing efficiency and swallowing safety) was determined with Cohen’s kappa coefficient *κ*.

#### Dysphagia for medication and motor complications

The MDS-UPDRS-Part-IV was used to characterize motor complications in the study participants. It consists of 6 ordinal items on typical motor complications, each scored from 0 to 4, contributing to a total score of 0 to 24. A linear regression model was used to predict motor complications according to the summarized score of the MDS-UPDRS-Part-IV with age and the dysphagia for medication score ranging from 0 to 32 (Methods-section “Swallowing-assessment”) as independent variables. Due to the violation of homoscedasticity of the residuals and normal distribution of the residuals, an additional wild-bootstrapping with 2000 iterations was performed to calculate an adjusted p-value for the dysphagia for medication score as independent variable.

#### Dysphagia for medication and type of tablet or capsule

The severity of impairment in swallowing efficiency and swallowing safety (Results-section “Classification of Dysphagia for Medication”) as well as the rating of subjective swallowing difficulty and subjective swallowing anxiety in the numeric rating scale were cross-tabulated depending on the type of tablet or capsule. The Friedman’s-test was used to investigate if there were statistically significant differences depending on the type of tablet or capsule. In case of significant differences, a post-hoc test was performed to pairwise compare each of the tablets and capsules. A Bonnferoni correction was applied in the post-hoc analysis to adjust the p-value for multiple testing.

#### Dysphagia for medication and OD for food and liquid

The severity of overall dysphagia for medication was cross-tabulated with OD for food and liquid and the PAS. The correlation between dysphagia for medication and OD for food and liquid and the PAS was calculated with Spearman’s rho correlation coefficient.

#### Swallowing-MDS-UPDRS-items as predictor of dysphagia for medications

Scores from item 2.3 (problems swallowing pills or eating meals, 0: normal—4: severe) and item 2.4 (troubles handling food, 0: normal—4: severe) of the MDS-UPDRS-Part-II were summed up and used to predict at least moderate overall dysphagia for medication (Methods-section “Swallowing-assessment”). A Receiver-Operating-Characteristic-analysis (ROC-analysis) was performed and the sum-score of the two swallowing-related MDS-UPDRS-items was implemented as a test variable to predict at least moderate overall dysphagia for medication as an outcome variable. Subsequently, an optimal cutoff-value for the prediction of dysphagia for medication was determined using the Youden-index. Sensitivity, specificity, positive predictive value, and negative predictive value were calculated for the respective cut-off-value.

### Reporting summary

Further information on research design is available in the Nature Research Reporting Summary linked to this article.

## Supplementary information

Supplementary Video 1 swallowing efficiency

Supplementary Video 2 swallowing safety

Reporting Summary

Legend in the supplementary material

## Data Availability

Due to data protection regulations according to the ethical vote for this study, personal patient data may only be made available to the study team. All relevant data have been provided in this manuscript in anonymized form. For questions regarding further data, the study team can be contacted so that—if possible—the requested data can be provided in an anonymized form (e.g., BL: Bendixruven.Labeit@ukmuenster.de or TW: Tobias.Warnecke@klinikum-os.de).
