# Supplementary material for: Dysphagia for medication in Parkinson’s disease
Source: NPJ Parkinsons Dis. 2022 Nov 12;8:156. doi: 10.1038/s41531-022-00421-9 (PMC9653428; doi:10.1038/s41531-022-00421-9)
Supplement: Supplementary file 4 — Legend in the supplementary material [file 41531_2022_421_MOESM4_ESM.docx]

**Legend in the supplementary material:**

**Supplementary video 1:** Illustration of the ordinal severity levels of impaird swallowing efficiency.

**Supplementary video 2:** Illustration of the ordinal severity levels of impaird swallowing safety.
